# Effects of cage and floor rearing system on the factors of antioxidant defense and inflammatory injury in laying ducks

**DOI:** 10.1186/s12863-019-0806-0

**Published:** 2019-12-30

**Authors:** Yang Zhang, Tiantian Gu, Yong Tian, Li Chen, Guoqin Li, Wei Zhou, Guofa Liu, Xinsheng Wu, Tao Zeng, Qi Xu, Guohong Chen, Lizhi Lu

**Affiliations:** 1 Jiangsu Key Laboratory for Animal Genetic, Breeding and Molecular Design, Yangzhou University, Yangzhou, 225009 Jiangsu China; 2 Institute of Animal Husbandry and Veterinary Medicine, Zhejiang Academy of Agricultural Sciences, Zhejiang, 310021 Hangzhou China; 3 Key Laboratory of Information Traceability for Agricultural Products, Ministry of Agriculture of China, Hangzhou, 310021 Zhejiang China; 4 Guiliu Animal Husbandry Company, Zhoukou, 450000 Henan China; 5 Key Laboratory of Animal Genetics, Breeding and Molecular Design of Jiangsu Province, Yangzhou University, Yangzhou, 225009 PR China

**Keywords:** Cage rearing, Duck, Antioxidant enzymes, Inflammatory cytokines

## Abstract

**Background:**

Cage-rearing in laying ducks, as a novel rearing system, not only fundamentally solves the pollution problem of the duck industry and improve bio-safety and product quality but also exhibits more benefits by implementing standardized production compared with the floor-rearing. Of course, this system also brings some welfare problems and stress injuries to layers due to lack of water environment and limited activities in the cages. However, the effects on the factors of antioxidant defense and inflammatory injury in the early cage stage are not well-understood.

**Results:**

In this study, eighty Shaoxing layers were reared on floor and in cages from 12 weeks of age. The ducks were caged 1, 2, 4, 7, and 10 days, the factors of antioxidant defense and inflammatory injury were investigated. The results showed that the caged ducks suffered liver injury to a certain extent when the ducks were just put into the cages. Analysis of antioxidant enzyme activities indicated that the different rearing system could not affect the change of antioxidant capacities, while the liver malondialdehyde (MDA) level was significant higher in the 2-d, 7-d, and 10-d ducks compared with the 1-d ducks during the change of days, while catalase (CAT) activity showed the opposite results. Additionally, quantitative real-time PCR (qRT-RCR) revealed that the relative mRNA levels of endoplasmic reticulum (ER) stress-related gene (CHOP and GRP78) were significantly upregulated in cage rearing ducks compared to that of the floor rearing ducks. Moreover, the mRNA levels of inflammatory cytokines including cycloxygenase-2 (COX-2), nitric oxide synthase (iNOS), Interleukin 1 beta (IL-1β), Interleukin 2 (IL-2) and Interleukin 6 (IL-6), were also increased significantly in caged layers.

**Conclusions:**

Taken together, although antioxidant defense has no obvious effect on cage stress, the stress levels of laying ducks vary greatly in the early cage stage, which not only caused liver tissue damage to some extent, but also resulted in increases in the expression of the factors of inflammatory injury. Therefore, we recommend that anti-stress agents should be added in the feed to alleviate the stress in the early cage stage.

## Background

The market demand for ducks is increasing, and the scale of breeding must be expanded. Many resources such as land area are severely limited, and free-range methods are insufficient to meet consumer demands. Therefore, it is necessary to increase the scale of cage rearing. Previous studies reported that growing laying ducks in cages mode can increase the egg production rate and feed-egg ratio and lower the egg breakage rate with no large effects on egg quality [1–3]. However, the influence of stress on the ducks grown in cages remains unclear.

All living organisms respond to different types of environmental stressors by synthesizing oxidative stress proteins through a variety of signaling pathways, such as the endoplasmic reticulum (ER) stress response [4]. ER stress is considered as an early or initial response of cells to stress or damage [5]. In chicken, the key regulator of the mRNA levels of the ER stress gene (GRP78) in under selenium-deficient stress was significantly elevated in the liver [6]. Additionally, the CHOP protein content is increased in pig exposed to high temperatures [7]. Oxidative stress can enhance the formation of reactive oxygen species (ROS), which induce lipid, protein, or DNA oxidation and enhance lipid peroxidation to cause oxidative injury [8]. The activities of superoxide dismutase (SOD) and catalase (CAT) and levels of malondialdehyde (MDA) are increased in the liver of broiler chickens during heat stress [9]. In the masseter muscles of psychologically stressed rats, glutathione peroxidase (GSH-Px) and CAT activities were decreased, and MDA content was increased after 3 and 5 weeks [10]. Exposure to various stress conditions can also induce inflammation. It has been reported that the level of interleukin (IL)-2 mRNA in humans is increased under cold stress [11]. Under chronic cold stress, IL-2 and IL-10 contents were increased in the spleen and bursa of Fabricius of chicken [12]. In addition, the expression of inflammatory factors such as iNOS and COX-2 are influenced by exposure to high temperature in the duck liver [13].

Cage-rearing of laying ducks, as a novel culture mode, is beneficial for improving land utilization, feed conversion and egg production rate while reducing the egg breakage rate. However, few studies have examined the influence of cage stress on laying ducks or molecular mechanism of cage stress. Thus, we compared the microstructures of the liver in ducks reared using two different breeding patterns by histopathological analysis and investigated the effect of cage stress on antioxidant enzyme activity (SOD, MDA, CAT, total antioxidant capacity (T-AOC), and GSH-PX) in the livers of ducks. Furthermore, the expression of ER stress-related genes (GRP78 and CHOP) and inflammatory factors (COX-2, iNOS, IL-1β, IL-2 and IL-6) were analyzed during cage stress. These data improve the understanding of the influence of cage stress, providing insight useful for the large-scale breeding of caged ducks.

## Methods

### Experimental design and management

The experimental animals were female Shaoxing ducks (*Anas platyrhynchos*) obtained from Guiliu Animal Husbandry Company (Henan, China), and were always raised on the ground before the period of trial. And then eighty 12-week-old ducks with similar body weights were randomly divided into two groups. The forty ducks were reared on the floor (RF ducks), the others were reared from the ground into the cage (RC ducks), which were in closed-end animal building. The RF ducks were fed in semi-enclosed house (0.375m^2^/per) and the RC ducks were raised in alone per cage (28 × 40 × 40 cm). All ducks kept at room temperature with flowing air for adaptation. The ducks were fed ad libitum with the same commercial formula diet (Henan Jinjing Biochemical Co., Ltd., Henan, China), which mainly contained corn, soya bean meal, rice bran, and wheat-middling throughout the study. The ducks were subjected to a standard light regimen of 17 h light (17 L:7D) throughout the experimental period. All experimental ducks were healthy and were not administered any antibiotic treatments during the experiment.

### Tissues collection

After 1, 2, 4, 7, and 10 days in the cage, five ducks from each group were randomly and sacrificed. Then the selected ducks at various times were immediately anesthetized with sodium pentobarbital (intraperitoneal injection: 150 mg/kg) and killed by exsanguination. The liver was collected from each duck and tissue samples were harvested and immediately snap-frozen in liquid nitrogen. Samples were stored at − 80 °C until analysis. Total RNA was extracted from all tissues with TRIZOL (Invitrogen, Carlsbad, CA, USA). And the other liver tissues were placed also in a 4% formaldehyde solution for histological analysis.

### Histopathologic analysis

For conventional histopathologic analysis, the liver tissue was fixed in 4% buffered formaldehyde and then embedded in paraffin, and 4-μm-thick serial sections were prepared and stained with hematoxylin and eosin according to standard protocols. The sections were analyzed under an Olympus light microscope (Tokyo, Japan) to detect evidence of injury. According to the degree of light to heavy lesions, a small amount or no lesion was considered as negative and given a score of 0; mild or small was scored as 1; moderate or medium was scored as 2; severe was scored as 3; very heavy was scored as 4.

### Antioxidant enzyme activities

The activities of SOD (U/mL), CAT(U/mL), T-AOC(U/mL), and GSH-PX(U/mL) and the MDA (nmol/mL) level were detected using superoxide dismutase assay kit (HY-60001), catalase assay kit (HY-60015), total antioxidant capacity assay kit (HY-60021), glutathione assay kit (HY-60006), and malondialdehyde assay kit (HY-60003), respectively. The experiments were performed according to the manufacturer’s protocols.

### RNA extraction and quantitative real-time PCR (qRT-RCR)

Total RNA was extracted from the liver tissue using Trizol (TAKARA, Dalian, China). The RNA was resuspended in RNase-free water, and the concentration and purity were measured using a NanoDrop Spectrophotometer (NanoDrop Technologies, Wilmington, DE, USA). RNA (1 μg) was mixed with gDNase (Toyobo, Osaka, Japan) for reverse transcription. The process included an initial step at 37 °C for 5 min, followed by incubation at 37 °C for 15 min, 50 °C for 5 min, and 98 °C for 5 min; cDNA was stored at − 80 °C for qRT-PCR. The primers used are listed in Additional file 1: Table S1. The qRT-PCR assay was carried out on a LightCycler 96 (Roche, Basel, Switzerland) with samples containing 10 μL SYBR Green Master Mix, 0.4 μL forward/reverse primer, 2 μL cDNA template and 7.2 μL RNA-free water (Vazyme, Nanjing, China). Reaction conditions included one cycle at 95 °C for 30 s, 40 cycles of 95 °C for 10 s and 60 °C for 30 s. Experiments detecting all genes were performed in triplicate and expression levels were assessed relative to duck β- actin as an internal standard. Relative gene expression levels were calculated using the 2^−ΔΔCt^ method.

### Statistical analysis

The comparisons among different rearing systems and different days in the cage were fulfilled using two-way ANOVA in SPSS 13.0 software (SPSS, Inc., Chicago, IL, USA). *P* level below 0.05 were considered to indicate statistical significance. All data were analyzed using GraphPad Prism 5.0 software (GraphPad, Inc., La Jolla, CA, USA) and the results are presented as the means ± S.E.

## Results

### Histopathology of the liver

**Fig. 1:**
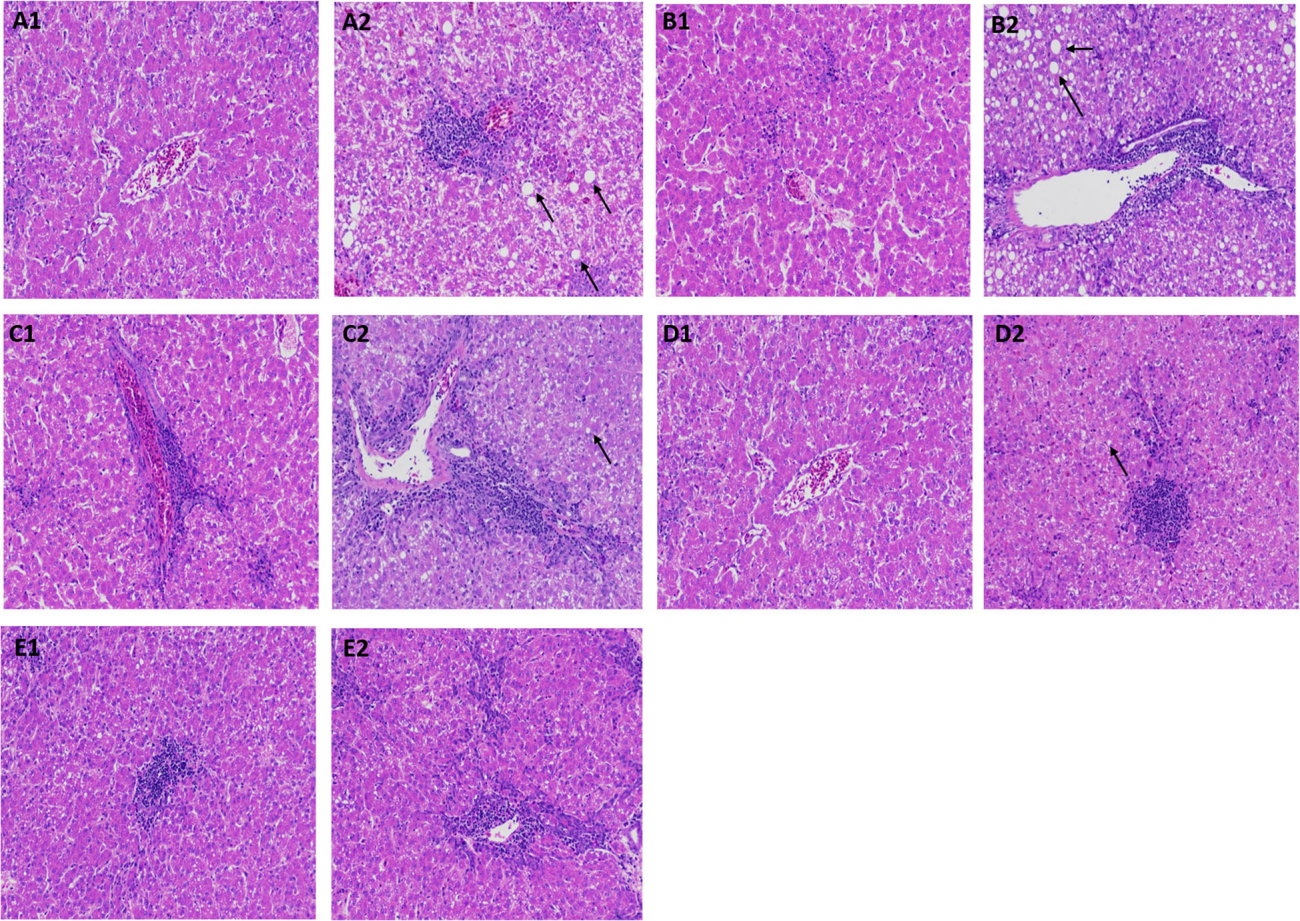
Histopathology of the liver. Hematoxylin and eosin staining of liver sections in the floor ducks (**a1-e1**) and cage ducks (**a2-e2**): **a, b, c, d, e** represented the days in the cage (1, 2, 4, 7, and 10 d, respectively). Black arrow represented cellular vacuolization

To determine the changes in liver tissue after cage stress, histological analysis was performed. The results indicated that RF ducks showed normal histological structure (Fig. 1a1-e1), while the livers of ducks reared in battery cages displayed some tissue injury corresponding to the time of cage stress (Fig. 1a2-e2). After 1 and 2 days of stress, the RC ducks showed severe liver injury, infiltration of inflammatory cells, and exudation of blood cells compared to the RF ducks (Fig. 1a2 and b2), indicating variable cellular vacuolization and hydropic degeneration in the liver in the early days of cage stress exposure. As the time of cage stress increased, liver injury got better after the 4-d, 7-d, and 10-d cage stress period (Fig. 1c2, d2, and e2).

### Antioxidant enzyme activity

**Fig. 2:**
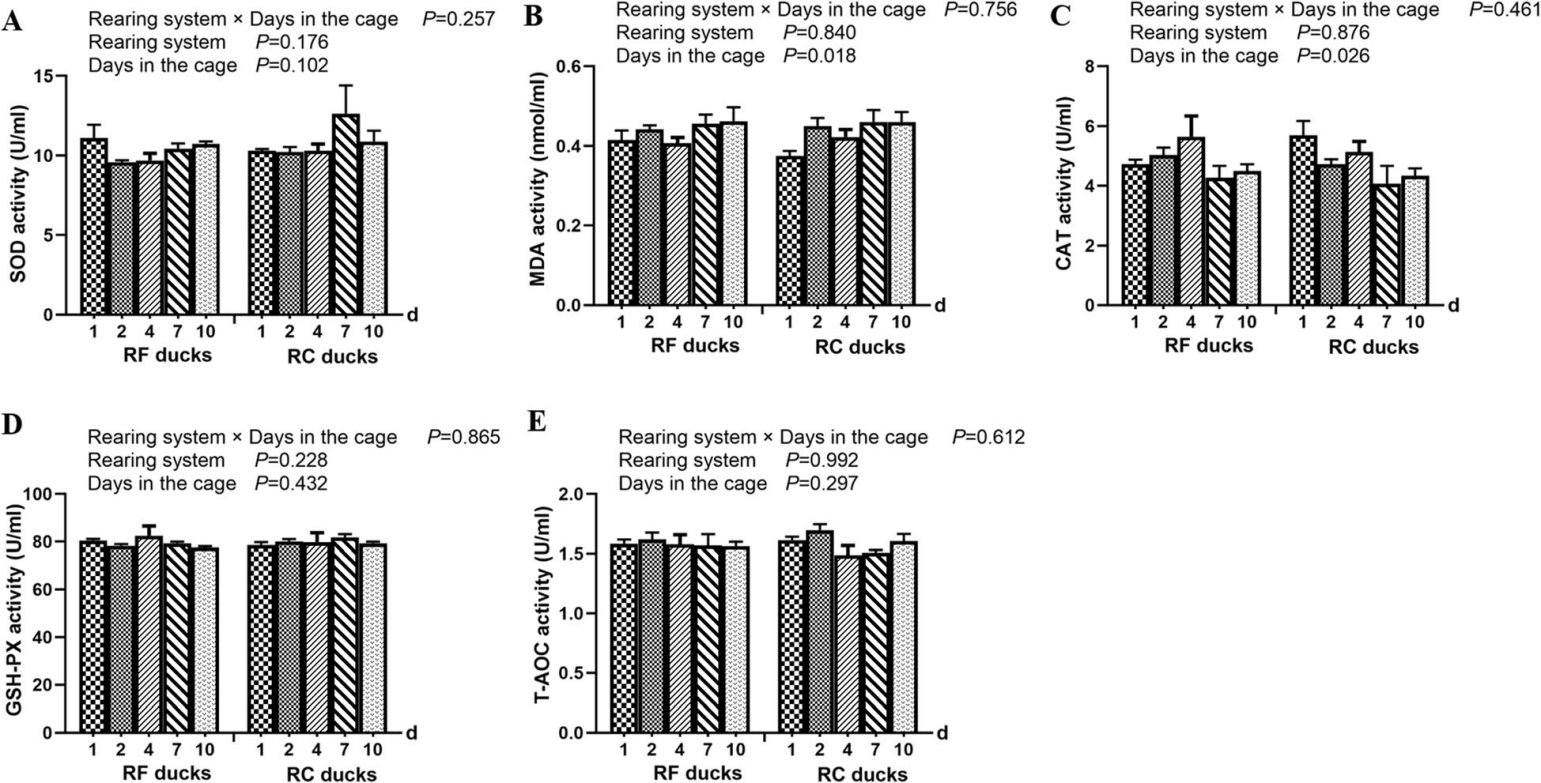
Effect of cage stress on antioxidant enzyme activity in the liver of Shaoxing ducks. Vertical bars represent the mean ± S.E. (*n* = 5). RF ducks = reared on the floor, RC ducks = reared in the cage

To further characterize the effect of cage stress on antioxidant capacity of Shaoxing ducks, the SOD, CAT, T-AOC, and GSH-PX activities and MDA level were measured. We observed that rearing systems did not cause significant changes on the activity of MDA, SOD, CAT, T-AOC, and GSH-PX (Fig. 2). On the other hand, the MDA level appeared a significant increase at 2 days and then showed a gently trend due to the change of days, besides, the MDA level was significant higher in the 2-d, 7-d, and 10-d ducks compared with the 1-d ducks during the change of days (Additional file 2: Table S2). While catalase (CAT) activity showed the opposite results that the CAT activity showed a great decreasing trend and then got a peaked significantly at 4 days.

### Expression of the liver CHOP and GRP78 in Shaoxing ducks

**Fig. 3:**
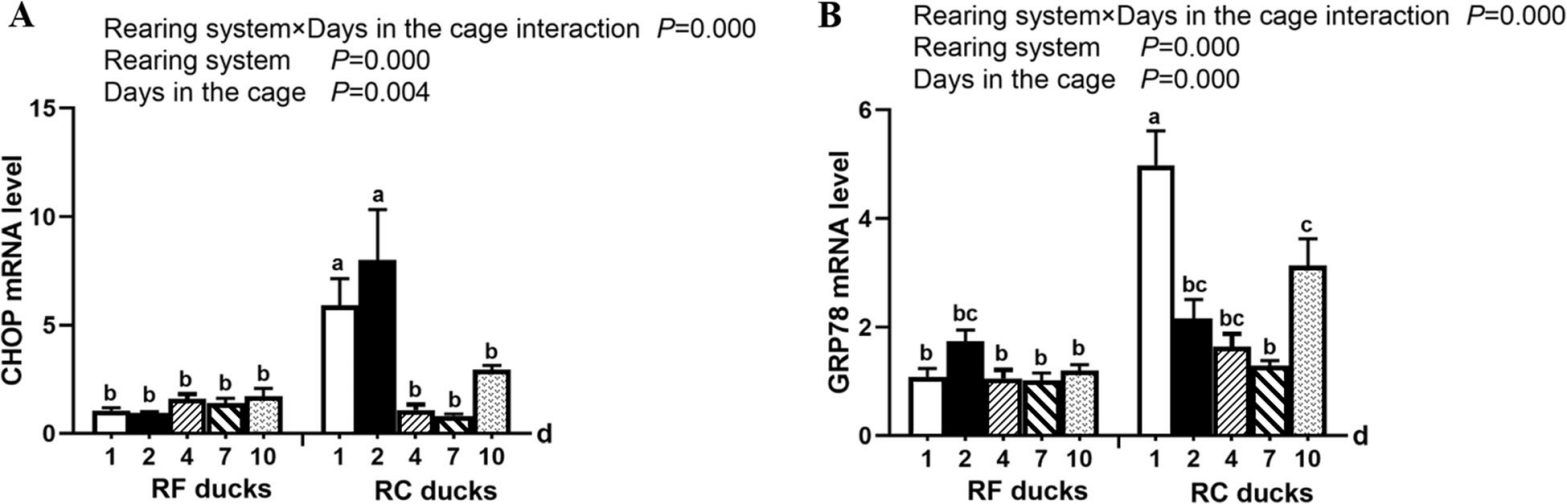
Effect of cage stress on mRNA expression of CHOP (**a**) and GRP78 (**b**) genes in the liver of Shaoxing ducks. Relative expression was normalized to the expression of β-actin. Vertical bars represent the mean ± S.E. (*n* = 5). RF ducks = reared on the floor, RC ducks = reared in the cage. ^a,b,c^Different superscripts within columns indicate means are significantly different. The labels 1, 2, 4, 7, and 10 d indicated the days in the cage

To evaluate the expression of ER stress response marker genes in the liver of Shaoxing ducks after cage stress, CHOP and GRP78 gene levels were measured by qRT-PCR. There was a significant interaction between factors (rearing system and days in the cage) for the CHOP and GRP78 level (Fig. 3). We found that CHOP mRNA was significantly upregulated in the RC ducks after 1 and 2 days of cage rearing, particularly in the RC ducks after 2 days, while the CHOP mRNA in RF ducks showed a gentle process (Fig. 3a). And comparing with the RF ducks, the CHOP mRNA expression in RC ducks was significantly increased in 1d and 2d. The mRNA expression of the GRP78 gene was significantly increased after 1 and 10 days of captivity, comparing with RF ducks (Fig. 3b).

### Expression of liver inflammatory cytokines in Shaoxing ducks

**Fig. 4:**
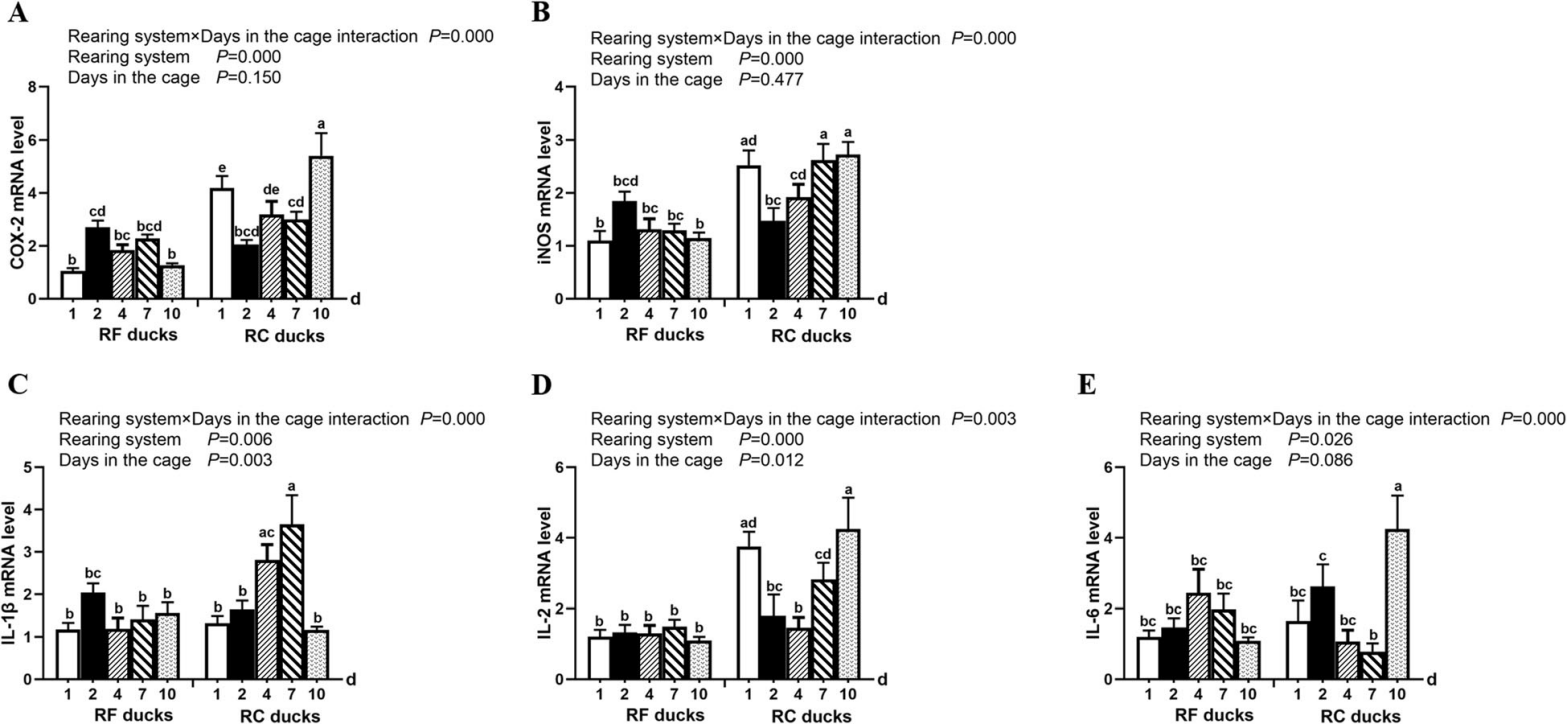
Effect of cage stress on mRNA expression of COX-2 (**a**), iNOS (**b**), IL-1β (**c**), IL-2 (**d**), and IL-6 (**e**) genes in the liver of Shaoxing ducks. Relative expression was normalized to that of β-actin. Vertical bars represent the mean ± S.E. (*n* = 5). RF ducks = reared on the floor, RC ducks = reared in the cage. ^**a,b,c,d,e**^Different superscripts within columns indicate means are significantly different. The labels 1, 2, 4, 7, and 10 d indicated the days in the cage

To determine the effect of cage stress on the mRNA levels of inflammatory genes in the liver. The significant interaction between factors (rearing system and days in the cage) for the COX-2, iNOS, IL-1β, IL-2 and IL-6 mRNA level was observed (Fig. 4). The iNOS mRNA expression in RF ducks appeared a gentle tendency while the iNOS mRNA level in RC ducks performed the tendency that first decreasing and then rising, especially in 10 days of cage stress. Besides, the iNOS mRNA levels in the RC ducks were significantly higher than that in the RF ducks, particularly in the 1-d, 4-d and 10-d (Fig. 4a). Meanwhile, the expression of COX-2 mRNA showed similar tendency with iNOS whether in the RF ducks or the RC ducks. The mRNA expression of COX-2 was significantly increased in the 1-d, 7-d, and 10-d cage stress group (Fig. 4b). Furthermore, the mRNA level of IL-1β was gradually upregulated, reaching a peak after 7 days in the RC ducks and the IL-1β mRNA levels in the RC ducks were significantly higher than that in the RF ducks, particularly in the 4-d and 7-d RC ducks (Fig. 4c). The IL-2 mRNA level was higher in the RC ducks than that in RF ducks, and was particularly significant in 1-d, 7-d and 10-d RC ducks. IL-2 mRNA levels showed a decreasing trend and then gradually increased during cage stress (Fig. 4d). Finally, the IL-6 mRNA expression in RC ducks showed no significant change until 10-d cage stress and IL-6 mRNA levels were significantly higher in the 10-d RC ducks than in the RF ducks (Fig. 4e).

## Discussion

Environmental stress is experience by most animals and can induce various responses involving the balance of the oxidant/anti-oxidant system, as well as cause oxidative damage to several tissues by altering the enzymatic and non-enzymatic antioxidant status and enhancing ROS production [14, 15]. The anti-oxidative enzyme system, including SOD, CAT, and GSH-Px, plays an important role in the first line of antioxidant defense and MDA acts as a general biomarker for biological oxidative stress [16]. Previous studies showed that the MDA level were upregulated significantly in chickens exposed to high temperatures [17]. Other studies demonstrated that the MDA content was increased by chronic heat stress [18]. While in the present study, the activities of anti-oxidative enzymes in different rearing systems did not change significantly, and the result was inconsistent with other studies [17, 18], which indicates that the body of ducks has a certain degree of adaptability to the external environment and the balance of the oxidant and antioxidant systems was cooperated to repair the ability of scavenge free radicals [19]. However, histological analysis of Shaoxing ducks revealed severe liver injury, infiltration of inflammatory cells, and exudation of blood cells, particularly in the 1-d and 2-d RC ducks. These results indicate that in the early cage stress period, short-term stress stimulation was sufficient to cause stress damage to tissues of the body [15]. The liver, as the most abundant organ of protein metabolism, is closely related to protein processing and also the target of attack in the endoplasmic reticulum [20]. Once the endoplasmic reticulum is attacked, the unfolded protein reaction in the endoplasmic reticulum is intensified, eventually leading to hepatocytes in the endoplasmic reticulum stress state, aggravating cell damage [1]. Interestingly, after 4 days of stress test, the liver of caged ducks got better, which indicated that the cage stress caused some damage to the liver, but did not exceed the range of adaptation of the body. Therefore, after 4 days of stress, the liver tissue structure has a tendency to return to normal due to self-regulation of the body.

A recent study has been revealed that oxidative stress induces continuous ER stress by interfering with oxidation of the internal environment of the ER, and thus the ER stress response may occur downstream of oxidative stress [6]. To determine the effects of cage rearing on ER stress in ducks, we investigated the expression levels of ER stress-related signaling molecules including CHOP and GRP78 in the liver tissues of normal and caged ducks. A previous study showed that GRP78 expression was increased by the pyrrolidine dithiocarbamate/Cu complex [21]. The expression GRP78 was significantly elevated in the spleen of chicken induced by heat stress [22]. In pig, the CHOP protein content was increased following exposure to the high temperature [7]. In our study, the expression levels of GRP78 and CHOP were significantly elevated in the liver of caged ducks, indicating that cage restraint can lead to ER stress in the liver of ducks.

Several studies reported that different environmental stressors influence immune system function to protect tissues from damage [23, 24]. iNOS and cytokines play essential roles in tissues during inflammatory processes. In broiler chicken, the mRNA expression of iNOS gene was significantly upregulated st cold temperatures [25], Cold temperatures also increased iNOS and COX-2 mRNA levels in Muscovy ducks [13]. In this study, the mRNA expression of COX-2 and iNOS was increased in Shaoxing ducks during cage stress, indicating activation of a host defense mechanism to regulate the inflammation process. Additionally, pro-inflammatory cytokines are important for recruiting immune cells to infection sites. In chicken, IL-1β and IL-6 cytokine mRNA levels were significantly upregulated following exposure to cold stress [24], with similar effects reported in humans [26, 27]. Cold stress induced the mRNA expression of IL-2 in the small intestine of broilers [28]. Consistent with these previous studies, our results showed that IL-1β, IL-2, and IL-6 mRNA levels were significantly increased trend, particularly in the late period of cage stress. This indicates that cage-restraint stress affects the liver function by regulating the expression of the cytokines.

## Conclusions

In summary, we provide insight into the influence of the early stress of captivity on Shaoxing duck. Expression analysis showed that the CHOP and GRP78 genes were significantly upregulated in the liver during cage stress, indicating that cage rearing induces the ER stress response. Although rearing systems did not cause significant changes on the activity of anti-oxidative enzymes, the caged ducks suffered liver injury to a certain extent. These results suggest that cage stress could lead to tissue damage and increase the expression levels of inflammatory factors during the cage stress period, which may provide useful information for performing cage rearing of egg-laying ducks on a large scale.

## Supplementary information

**Additional file 1: Table S1.** The primers of expressed genes detected in the study.

**Additional file 2: Table S2.** Main effect of rearing system and days in the cage on SOD, MDA, CAT, GSH-PX and T-AOC activity

## Data Availability

The data sets supporting the results of this article are included within the article and its additional file.

## Abbreviations

CAT: Catalase
COX-2: Cycloxygenase-2
ER: Endoplasmic reticulum
ES: Environmental stress
GSH-Px: glutathione peroxidase
IL: Interleukin
IL-1β: Interleukin 1 beta
IL-2: Interleukin 2
IL-6: Interleukin 6
iNOS: Nitric oxide synthase
MDA: Malondialdehyde
qRT-RCR: Quantitative real-time PCR
RC ducks: Reared in the cage
RF ducks: Reared on the floor
ROS: Reactive oxygen species
SOD: Superoxide dismutase;
T-AOC: Total antioxidant capacity
UPR: Unfolded protein response
