# Supplementary material for: Effects of cage and floor rearing system on the factors of antioxidant defense and inflammatory injury in laying ducks
Source: BMC Genet. 2019 Dec 30;20:103. doi: 10.1186/s12863-019-0806-0 (PMC6937681; doi:10.1186/s12863-019-0806-0)
Supplement: Supplementary file 1 — Additional file 1: Table S1. The primers of expressed genes detected in the study. [file 12863_2019_806_MOESM1_ESM.docx]

Table S1. Primers used in the study.

| Primer name | Primer sequence(5’→3’) | Annealing temperature (°C) | Application |
| --- | --- | --- | --- |
| qCHOP-F | AAGAGGGGCAGTGGTGT | 60 | qRT-PCR |
| qCHOP-R | TAGGTCTGGCGGAGGTT |  |  |
| qGRP78-F | TTACTGTGCCAGCCTACTT | 60 | qRT-PCR |
| qGRP78-R | GGATGTTCTTCTCACCCTCT |  |  |
| qCOX-2-F | CACGCTCTGATTGTTGCC | 60 | qRT-PCR |
| qCOX-2-R | AGGATTTGTAGGGATGGG |  |  |
| qiNOS-F | CCACCAGGAGATGTTGAATATGTC | 60 | qRT-PCR |
| qiNOS-R | AGGATTTGTAGGGATGGG |  |  |
| qIL-1β-F | TGGGCATCAAGGGCTACAAG | 60 | qRT-PCR |
| qIL-1β-R | GCTGTCGATGTCCCTCATGAC |  |  |
| qIL-2-F | GCCAAGAGCTGACCAACTTC | 60 | qRT-PCR |
| qIL-2-R | ATCGCCCACACTAAGAGCAT |  |  |
| qIL-6-F | TTCGACGAGGAGAAATGCTT | 60 | qRT-PCR |
| qIL-6-R | CCTTATCGTCGTTGCCAGAT |  |  |
| β-actin-F | ATGTCGCCCTGGATTTCG | 60 | qRT-PCR |
| β-actin-R | CACAGGACTCCATACCCAAGAA |  |  |
