# Supplementary material for: Effects of cage and floor rearing system on the factors of antioxidant defense and inflammatory injury in laying ducks
Source: BMC Genet. 2019 Dec 30;20:103. doi: 10.1186/s12863-019-0806-0 (PMC6937681; doi:10.1186/s12863-019-0806-0)
Supplement: Supplementary file 2 — Additional file 2: Table S2. Main effect of rearing system and days in the cage on SOD, MDA, CAT, GSH-PX and T-AOC activity [file 12863_2019_806_MOESM2_ESM.docx]

**Table S2.** Main effect of rearing system and days in the cage on SOD, MDA, CAT, GSH-PX and T-AOC activity

| Main Effect |  | SOD  (U/ml) | MDA  (nmol/ml) | CAT  (U/ml) | GSH-PX  (U/ml) | T-AOC  (U/ml) |
| --- | --- | --- | --- | --- | --- | --- |
| rearing system | RF ducks | 10.289±1.034 | 0.437±0.048 | 4.846±0.978 | 10.289±1.034 | 1.581±0.135 |
|  | RC ducks | 10.828±1.762 | 0.434±0.056 | 4.793±1.010 | 10.828±1.762 | 1.576±0.130 |
| days in the cage | 1 | 10.665±1.124 | 0.393±0.041^b^ | 5.268±0.932^ac^ | 10.665±1.124 | 1.600±0.619 |
|  | 2 | 9.945±0.616 | 0.447±0.034^ac^ | 4.859±0.437^abc^ | 9.945±0.616 | 1.656±0.110 |
|  | 4 | 9.968±0.927 | 0.415±0.037^bc^ | 5.394±1.194^ac^ | 9.968±0.927 | 1.532±0.175 |
|  | 7 | 11.420±2.516 | 0.459±0.056^ac^ | 4.170±1.079^b^ | 11.420±2.516 | 1.537±0.149 |
|  | 10 | 10.824±1.086 | 0.462±0.058^a^ | 4.406±0.491^b^ | 10.824±1.086 | 1.585±0.108 |
| ***P*-Value**（Two-Way ANOVA） | | | |  |  |  |
| rearing system | | 0.176 | 0.840 | 0.876 | 0.228 | 0.992 |
| days in the cage | | 0.102 | 0.018 | 0.026 | 0.432 | 0.297 |
| rearing system * days in the cage | | 0.257 | 0.756 | 0.461 | 0.865 | 0.612 |

Note: RF ducks= reared on the floor, RC ducks= reared in the cage.
